# A Simple and Effective Flow Cytometry-Based Method for Identification and Quantification of Tissue Infiltrated Leukocyte Subpopulations in a Mouse Model of Peripheral Arterial Disease

**DOI:** 10.3390/ijms21103593

**Published:** 2020-05-19

**Authors:** Konda Kumaraswami, Natallia Salei, Sebastian Beck, Stephan Rambichler, Anna-Kristina Kluever, Manuel Lasch, Lisa Richter, Barbara U. Schraml, Elisabeth Deindl

**Affiliations:** 1 Walter-Brendel-Centre of Experimental Medicine, University Hospital, LMU Munich, 81377 Munich, Germany; Kumaraswami.Konda@med.uni-muenchen.de (K.K.); Natallia.Salei@med.uni-muenchen.de (N.S.); s.beck1@campus.lmu.de (S.B.); stephan.rambichler@med.uni-muenchen.de (S.R.); annakluever97@gmail.com (A.-K.K.); manuel_lasch@gmx.de (M.L.); barbara.schraml@med.uni-muenchen.de (B.U.S.); 2 Biomedical Center, Institute of Cardiovascular Physiology and Pathophysiology, LMU Munich, 82152 Planegg-Martinsried, Germany; 3 Department of Otorhinolaryngology, Head & Neck Surgery, University Hospital, LMU Munich, 81377 Munich, Germany; 4 Core Facility Flow Cytometry, Biomedical Centre, LMU Munich, 82152 Planegg-Martinsried, Germany; l.richter@med.uni-muenchen.de

**Keywords:** peripheral arterial disease, arteriogenesis, shear stress, flow cytometry, leukocytes, immunohistology, inflammation, collateral artery growth

## Abstract

Arteriogenesis, the growth of a natural bypass from pre-existing arteriolar collaterals, is an endogenous mechanism to compensate for the loss of an artery. Mechanistically, this process relies on a locally and temporally restricted perivascular infiltration of leukocyte subpopulations, which mediate arteriogenesis by supplying growth factors and cytokines. Currently, the state-of-the-art method to identify and quantify these leukocyte subpopulations in mouse models is immunohistology. However, this is a time consuming procedure. Here, we aimed to develop an optimized protocol to identify and quantify leukocyte subpopulations by means of flow cytometry in adductor muscles containing growing collateral arteries. For that purpose, adductor muscles of murine hindlimbs were isolated at day one and three after induction of arteriogenesis, enzymatically digested, and infiltrated leukocyte subpopulations were identified and quantified by flow cytometry, as exemplary shown for neutrophils and macrophages (defined as CD45^+^/CD11b^+^/Ly6G^+^ and CD45^+^/CD11b^+^/F4/80^+^ cells, respectively). In summary, we show that flow cytometry is a suitable method to identify and quantify leukocyte subpopulations in muscle tissue, and provide a detailed protocol. Flow cytometry constitutes a timesaving tool compared to histology, which might be used in addition for precise localization of leukocytes in tissue samples.

## 1. Introduction

Peripheral occlusive diseases, including atherosclerosis, myocardial infarction and stroke, are a wide spectrum of arterial-based pathologies, reported as one of the leading causes of death worldwide [1]. Currently, the therapeutic options mainly consist of surgical interventions such as balloon dilatation and stent implantation, as well as bypass surgery [2]. However, the body possesses an endogenous mechanism to compensate for stenosis and obstructions by forming natural bypasses. This process is called arteriogenesis [3]. Understanding the mechanisms of arteriogenesis can help to design new therapeutic options to accelerate the process of arteriogenesis in patients with the final aim of minimizing invasive interventions and avoiding amputation.

Previous studies, predominantly performed on murine hindlimb models [4], showed that arteriogenesis essentially depends on the recruitment of leukocytes to the perivascular space, where these cells subsequently supply growth factors for collateral remodeling [5,6]. At present, immunohistochemical (IHC) staining is the technique of choice for analyzing and quantifying the leukocyte subpopulations involved in this process. However, this method is very time-consuming. Analysis by flow cytometry offers an elegant and time-saving alternative to IHC especially in regard to the quantification of leukocytes.

## 2. Materials

### 2.1. Mice

C57BL/6J, 8–12 weeks old male mice from Charles River were used. Animal studies were conducted in compliance with ethical standards and with the approval of the Government of Upper Bavaria, Germany. Mice were anesthetized with the standard solution of fentanyl (0.05 mg/kg), midazolam (5 mg/kg) and medetomidine (0.5 mg/kg) applied subcutaneously (s.c.). To antagonize the narcosis, a combination of naloxone (1.2 mg/kg), flumazenile (0.5 mg/kg) and atipamezole (2.5 mg/kg) was applied s.c. To prevent postsurgical pain, mice were treated with buprenorphine (0.05 mg/kg) every 12 h until day 3.

### 2.2. Reagents

- Fentanyl (CuraMED Pharma, Karlsruhe, Germany)
- Midazolam (Ratiopharm GmbH, Ulm, Germany)
- Medetomidine (Pfizer Pharma, Berlin, Germany)
- Buprenorphine (Reckitt Benckiser Healthcare, London, UK)
- Naloxone (Inresa Arzneimittel GmbH, Freiburg, Germany)
- Flumazenile (Inresa Arzneimittel GmbH, Freiburg, Germany)
- Atipamezole (Zoetis, Berlin, Germany)
- Phosphate buffered saline (PBS, Sigma-Aldrich, Taufkirchen, Germany, cat. D8537)
- 1% fetal bovine serum (FBS, Sigma-Aldrich, Taufkirchen, Germany, cat. F7524)
- Ethylenediaminetetraaceticacid (EDTA, Invitrogen, Waltham, MA, USA, cat. 15575020)
- 0.02% sodium azide (Sigma-Aldrich, Taufkirchen, Germany, cat. 71289)
- Collagenase IV (10,000 U/ml, Worthington, Freehold, NJ, USA, cat. CLS4)
- DNase I (20 mg/ml, Roche, Penzberg, Germany, cat. 11284932001)
- RPMI 1640 medium (Gibco, Dubline, Ireland, cat. 31870-074)
- Percoll (GE Healthcare, Chicago, IL, USA, cat. 17-0891-01)
- Hank’s Balanced Salt solution (HBSS, Sigma-Aldrich, Taufkirchen, Germany, cat. H9394-500ML)
- Mounting medium (Thermo Fisher Scientific, Schwerte, Germany, cat. TA-030-FM)
- 4% paraformaldehyde (PFA, Morphisto, Frankfurt am Main, Germany, cat. 1176.00500)

The following antibodies were used for flow cytometry:

- PE/Cy7 anti-mouse CD45.2 (Biolegend, Eching, Germany, cat. 109829, clone: 104, 1/300 dilution)
- BUV737 anti-CD11b (BD Biosciences, Heidelberg, Germany, cat. 564443, clone: M1/70, 1/800 dilution)
- Brilliant Violet 785 anti-mouse F4/80 (Biolegend, Eching, Germany, cat. 123141, clone: BM8, 1/100 dilution)
- PerCP/Cy5.5 anti-mouse Ly6G (Biolegend, Eching, Germany, cat. 127615, clone: 1A8, 1/300 dilution)
- Anti-CD16/32 (BD Biosciences, Heidelberg, Germany, cat. 553142, clone: 2.4G2, 1/300 dilution)
- DAPI (4’, 6-diamidin-2-phenylindole, Sigma-Aldrich, Taufkirchen, Germany, Cat. D9542-5MG, 1/1000 dilution)

The following antibodies were used for immunohistological staining:

- Neutrophils (Ly6G, eBiosciences, San Diego, CA, USA, cat. 16-9668-82, 1/50 dilution)
- Macrophages (CD68-AF488, Abcam, Cambridge, UK, cat. ab201844, 1/100 dilution)
- Endothelial cells (CD31-AF647, BioLegend, cat. 102516, 1/50 dilution)
- Anti-mouse AF546 secondary antibody (Invitrogen, Waltham, MA, USA, cat. A11081, 1/200 dilution)

The following equipment were employed for measurements:

- Bijou sample container (Sigma-Aldrich, Taufkirchen, Germany, cat. Z645338-700EA)
- Scissors (Fine Science Tools, Heidelberg, Germany, cat. 812005-10)
- Falcon tubes 15mL and 50mL (Thermo Fisher Scientific, cat. 10468502/10788561)
- Falcon cell strainer (Thermo Fisher Scientific, cat. 352350)
- 96 well plate (V-bottom, Costar, Schwerte, Germany, cat. 3897)

### 2.3. Flow Cytometry

Flow cytometry measurements were performed using BD LSRFortessa (BD Biosciences) and analyzed on BD FACSDiva v8.0 and FlowJo-v10 software (BD Biosciences).

### 2.4. Immunohistochemical Analysis

Images were acquired using a Zeiss epifluorescent microscope (Carl Zeiss Microscopy GmbH, Munic, Germany) and analyzed using AxioVisionRel 4.8 software (Carl Zeiss Microscopy GmbH). 

## 3. Methods

### 3.1. Reagent Preparation

For flow cytometric cell analysis, the following reagents were prepared: FACS buffer, enzyme solution, and three gradient Percoll solutions.

The FACS buffer was prepared as a solution of PBS, 1% fetal bovine serum, 2.5 mM EDTA and 0.02% sodium azide.

Two-fold concentrated enzyme solution was prepared using a solution of collagenase IV and DNase I at a concentration of 400 U/ml (collagenase IV) and 0.4 mg/mL (DNase I) in RPMI-1640, respectively.

For isotonic Percoll stock solution, 45 mL Percoll and 5 mL 10× PBS were mixed. The solution was then prepared in three concentrations (70%, 37% and 30%). For 70% and 30% Percoll, the prepared Percoll stock solution was diluted with Hank’s Balanced Salt solution with phenol red. For 37% Percoll, the prepared Percoll stock solution was diluted with PBS. Different diluents were used for easy recognition of phases.

### 3.2. Surgical Procedure for Artery Ligation and Tissue Collection

The murine hindlimb ischemia model is a widely used mouse model to study arteriogenesis in detail [7]. The femoral artery of the right leg is ligated, while the left leg is sham operated and serves as an internal control [8]. After femoral artery ligation (FAL), the blood flow is redirected into preexisting arteriolar connections, which thereupon experience increased shear stress [9,10], and results in perivascular recruitment of leukocytes supplying growth factors and cytokines to the growing vessels [5,6].

**Figure 1:**
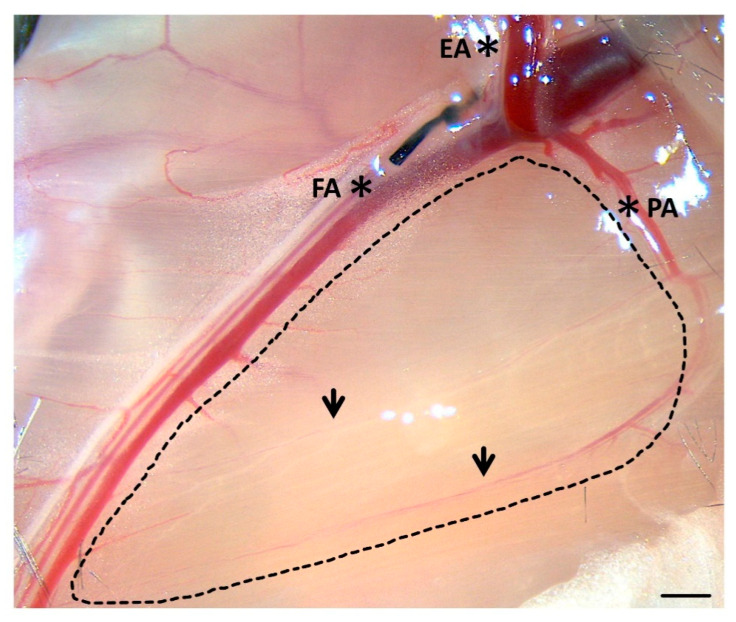
Tissue sampling on the right hindlimb of a C57BL/6J mouse. The part of the M. adductor containing the collaterals (arrows) was extracted along the dashed line. The collaterals connect the profunda femoris artery (PA) to the femoral artery (FA). The epigastric artery (EA) serves as orientation for the ligation. Scale bar 1 mm.

Depending on the experimental setup, the muscle tissue, in which collaterals are located, can be removed at various time points after arterial occlusion. To analyze the numbers and subpopulations of recruited leukocytes, we have chosen two time points: day 1 and day 3 after femoral artery ligation. For this purpose, the hindlimbs of the mice were perfused with FACS buffer via an aortic catheter (inserted distal to the outlet of the renal arteries). Thereafter, the adductor muscle of the mouse was removed along the dashed line as shown in Figure 1, whereby the femoral artery (FA) and profunda femoris artery (PA) were omitted from isolation. The muscle section was immediately rinsed with FACS buffer and further processed on ice.

### 3.3. Stepwise Preparation of a Single Cell Suspension from Collected Tissues

To prepare a single cell suspension for flow cytometric analysis, collected tissue samples were processed using the following protocol:

1. Place the muscle tissue (isolated from one hindlimb) in a bijou vial containing 1 mL of RPMI medium and finely cut the muscle with microsurgical scissors.
2. Add 1 mL of enzyme solution to digest the tissue (final concentration of collagenase IV and DNase I are 200 U/mL and 0.2 mg/mL, respectively).
3. Incubate the tube in a laboratory shaker for 1 h at 37 °C with 120 rpm.
4. Filter the digested tissue through a 70 µm cell strainer and fill up to 50 mL with FACS buffer.
5. Centrifuge the cells for 5 min at 4 °C with 420 g, then carefully discard the supernatant.
6. Resuspend the cell pellet in 4 mL of 70% Percoll.
7. Gently cover the solution with 4 mL of 37% Percoll followed by 1 mL of 30% Percoll.
8. Centrifuge for 30 min at room temperature with 940 g. Centrifuge acceleration and braking should be set to minimum to avoid disintegration of the Percoll gradient.
9. Remove the interphase cells located between the 70% and 37% phases, transfer them to a 15 mL Falcon tube and fill up to 15 mL with the FACS buffer.
10. Centrifuge the cells for 5 min at 4 °C with 420 g, then discard the supernatant.
11. Resuspend the cells in 100 µl FACS buffer for further processing (if the leukocyte subpopulation of interest is not frequent, cells from mice of the same group can be pooled).

### 3.4. Cell Staining for Flow Cytometry

For cell staining, the single cell suspension that was prepared from collected tissues was processed using the following protocol:

1. Transfer the cell suspension to a 96-well V-bottom plate.
2. Centrifuge the cells for 5 min at 4 °C with 420 g, then discard supernatant by decantation.
3. Resuspend the cells in 50 μL Fc-Block (FACS buffer containing 1.6 µg/mL anti-CD16/32).
4. Incubate for 10 min at 4 °C.
5. Add 50 µL of a two-fold concentrated staining solution (50μL FACS buffer with anti-CD45.2, anti-CD11b, anti-Ly6G and anti-F4/80).
6. Incubate the solution for 20 min at 4 °C in the dark.
7. Centrifuge for 5 min at 4 °C with 420 g, then discard the supernatant.
8. Resuspend the cells twice in 200 μL FACS buffer.
9. Centrifuge for 5 min at 4 °C with 420 g, then discard the supernatant.
10. Resuspend the stained cells in 100 μL FACS buffer.
11. Add 1 μL of 20 µg/mL DAPI in PBS to the cell suspension just before the measurement.
12. Analyze the cell suspension using a flow cytometer.

### 3.5. Immunohistochemical Staining

Adductor muscle tissue samples were collected after perfusion as previously described [8]. For staining, 10 µm thick frozen sections were used. The following protocol for staining was used:

1. Fix the sections in 4% PFA for 5 min.
2. Wash the sections in PBS for 5 min, repeat twice.
3. Add blocking buffer (1% BSA in PBST) to sections to prevent nonspecific binding.
4. Incubate for 30 min at room temperature.
5. Dilute the primary antibodies against all leukocytes, macrophages, neutrophils and endothelial cells in blocking buffer.
6. Incubate the sections in a humidified dark chamber at 4 °C overnight.
7. Wash the sections in PBS for 5 min, repeat twice.
8. Incubate the sections with anti-mouse AF546 secondary antibody (only for CD45 and Ly6G) for 1 h at room temperature.
9. Wash the sections in PBS for 5 min, repeat twice.
10. Follow the overnight incubation with antibody against endothelial cells.
11. Wash the sections in PBS for 5 min, repeat twice.
12. Incubate the sections with DAPI for 10 min at room temperature.
13. Wash the sections in PBS for 5 min, repeat twice.
14. Fix the sections with mounting medium, then store at 4 °C.

### 3.6. Statistical Analyses

Statistical analysis was performed using GraphPad Prism v. 8 software. Data are mean ± standard error of the mean (S.E.M.). Significances between two time points were calculated by unpaired student’s *t*-test. *p* < 0.05 was considered statistically significant.

## 4. Results

**Figure 2:**
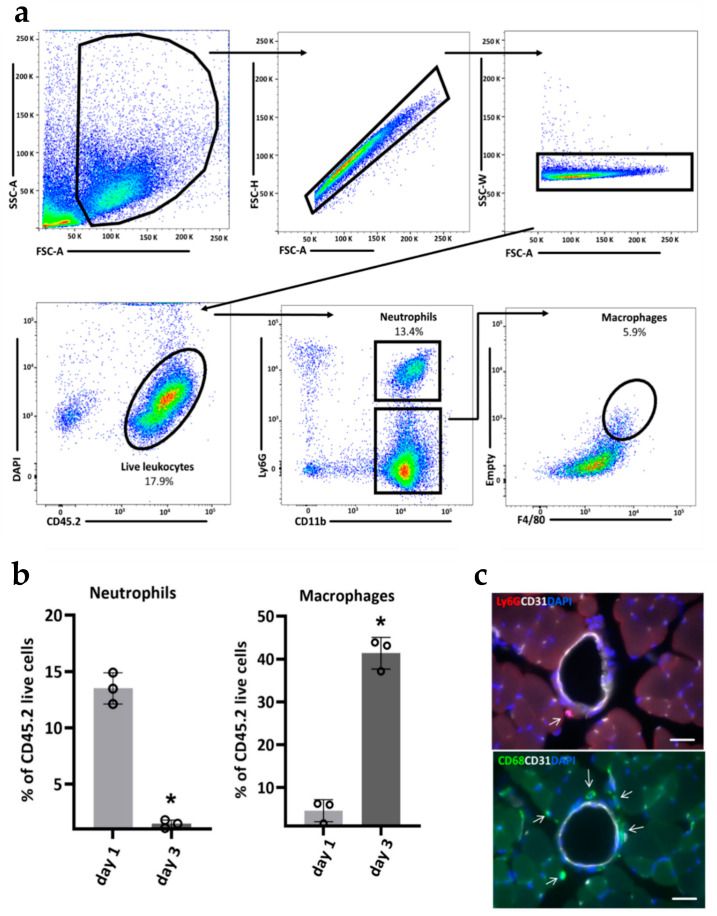
Gating strategy of flow cytometry and immunohistological analyses. (**a**) Sequential gating strategy for the identification of neutrophils (CD45.2^+^CD11b^+^Ly6G^+^) and macrophages (CD45.2^+^CD11b^+^F4/80^+^) in the murine adductor muscle containing growing collateral arteries at day 1 after FAL. (**b**) Bar graphs showing the frequencies of neutrophils and macrophages at day 1 and day 3 after FAL. *n* = 3 mice/group, data are represented as mean ± S.E.M., * *p* < 0.05 from unpaired student’s *t*-test. (**c**) Representative immunohistochemical stains demonstrate the presence of neutrophils (Ly6G^+^) and macrophages (CD68^+^) (indicated by arrows) in the perivascular space of growing collaterals in the adductor muscle of a mouse 3 days after FAL. Collaterals were stained with an endothelial marker (CD31) and nuclei with DAPI. Scale bar 20 µm.

Figure 2a shows the gating strategy used for the identification of leukocyte subpopulations, i.e., neutrophils and macrophages. First, we gated on leukocytes based on forward scatter (FSC) and side scatter (SSC) properties and excluded small cell debris. Next, we gated on single cells by plotting the height against the area for forward scatter (FSC-H vs FSC-A), and the width for side scatter against the area for forward scatter (SSC-W vs FSC-A). After doublets exclusion, we distinguished live cells (identified as DAPI negative) CD45.2^+^ leukocytes. Within live leukocytes we identified CD11b^+^ and Ly6G^+^ neutrophils, CD11b^+^ and F4/80^+^ macrophages. The proportion of neutrophils and macrophages were quantified on day 1 and 3 after ligation (Figure 2b). IHC staining demonstrated a localization of neutrophils and macrophages in the perivascular space of growing collateral arteries in the adductor muscle of mice as exemplary shown for day 3 after induction of arteriogenesis by FAL (Figure 2c).

## 5. Conclusions

Flow cytometry is an effective and high-throughput method to analyze and quantify infiltrated leukocyte subpopulations in muscle tissue. If necessary, it can be supplemented by IHC studies on the localization of those leukocytes. 

The protocol includes some limitations. Inadequate perfusion of the animals may be associated with residuals of leukocyte in vessels, resulting in inconsistent data. Poor enzymatic muscle digestion may lower the cell yield. In addition, cell preparation that involves washing and centrifugation might lead to cell loss of an unknown extent. 

However, when accurately performed, flow cytometry is an elegant method that can be used to complement results obtained from histological analysis, especially those pertaining to cell differentiation and cell counting. Further, flow cytometric analysis gives the possibility to calculate the absolute number of leukocytes per mg muscle tissue by the addition of counting beads to the cell suspension. With the increasing functions of flow cytometers, the further development of tissue extraction methods as well as the capacity to stain various markers on cell surfaces and in the cell interior, the application spectrum of flow cytometry is extremely broad.

## Abbreviations

IHC	immunohistochemical
FACS	Fluorescence Activated Cell Sorting
FA	femoral artery
FAL	femoral artery ligation
PA	profunda femoris artery
EA	epigastric artery
FCS	forward scatter
SSC	side scatter
